# Distribution of Killer-Cell Immunoglobulin-Like Receptor Genes and Combinations of Their Human Leucocyte Antigen Ligands in 11 Ethnic Populations in China

**DOI:** 10.3390/cells8070711

**Published:** 2019-07-12

**Authors:** Yufeng Yao, Lei Shi, Jiankun Yu, Shuyuan Liu, Yufen Tao, Li Shi

**Affiliations:** Institute of Medical Biology, Chinese Academy of Medical Sciences & Peking Union Medical College, Kunming 650118, China

**Keywords:** KIR, KIR–HLA pairs, ethnic populations in China

## Abstract

The aim of this study was to analyze the distribution of killer-cell immunoglobulin-like receptor (KIR) genes and their human leucocyte antigen (HLA) ligand combinations in different original ethnic populations in China, and thus, to provide relevant genomic diversity data for the future study of viral infections, autoimmune diseases, and reproductive fitness. A total of 1119 unrelated individuals from 11 ethnic populations—including Hani, Jinuo, Lisu, Nu, Bulang, Wa, Dai, Maonan, Zhuang, Tu, and Yugu—from four original groups, were included. The presence/absence of the 16 KIR loci were detected, and the KIR gene’s phenotype, genotype, and haplotype A and B frequencies, as well as KIR ligand’s HLA allotype and KIR–HLA pairs for each population, were calculated. Principal component analysis and phylogenetic trees were constructed to compare the characteristics of the KIR and KIR–HLA pair distributions of these 11 populations. In total, 92 KIR genotypes were identified, including six new genotypes. The KIR and its HLA ligands had a distributed diversity in 11 ethnic populations in China, and each group had its specific KIR and KIR–HLA pair profile. The difference among the KIR–HLA pairs between northern and southern groups, but not among the four original groups, may reflect strong pressure from previous or ongoing infectious diseases, which have a significant impact on KIR and its HLA combination repertoires.

## 1. Introduction

Segregated in different chromosomes, 6p21 and 19q13.4, human leucocyte antigen (HLA) and killer-cell immunoglobulin-like receptor (KIR) genes, respectively, exhibit diverse polymorphisms and their molecular expressions interact with each other as receptor ligands to ensure the proper role of nature killer (NK) cells in modulating an immune response [1,2]. Several studies on the coinheritance of these two genetic systems have indicated that carrying the appropriate KIR–HLA combination is important for human survival [3,4]. During human migration outward from Africa and the successive colonization worldwide, the cooperative KIR haplotypes and activating KIR–HLA pairs are important for humans to adapt to quickly changing environments and to increase population reproduction [5,6].

HLA polymorphism has been well studied in worldwide populations, which makes it a genetic marker for tracing a population’s origin, migration, and admixture [7,8]. Compared to HLA, KIR genes show polymorphisms, both at content and allelic levels. Among 16 identified KIR genes, *2DL1, 2DL2, 2DL3, 2DL5, 3DL1, 3DL2*, and *3DL3* belong to inhibitory KIR genes, while *2DS1, 2DS2, 2DS3, 2DS4, KIR2DS5*, and *3DS1* belong to activating KIR genes, and *KIR2DL4* has both inhibitory and activating capacities [9]. Four framework KIR genes—*3DL3, 3DP1, 2DL4*, and *3DL2*—which are located from the centromeric to the telomeric region, respectively, are observed consistently in almost all individuals [10]. On the basis of gene content, KIRs are divided into two haplotype groups: A and B. The A haplotype has a fixed gene content (*3DL3-2DL3-2DL1-3DP1-2DL4-3DL1-2DS4-3DL2*) and an activating *2DS4.* Four inhibitory KIRs (*2DL1*, *2DL3*, *3DL1*, and *3DL2*) are specific for four major HLA class I ligands (*C2*, *C1*, *Bw4*, and *A3/A11*, respectively) [11]. In contrast, haplotype B is variable both in the numbers and combinations of KIR genes and comprises several genes (*2DL2*, *2DL5*, *2DS1*, *2DS2*, *2DS3*, *2DS5*, and *3DS1*) that do not exist in the A haplotype. Among them, *2DS1* binds to *HLA-C2* but with a low affinity; *3DS1* may bind to the *HLA-Bw4* allotype, especially for *Bw4-80T*; and *2DS2* may bind to *HLA-A11* [12,13,14]. KIR genes, genotypes, haplotypes, and KIR–HLA pairs show a diverse distribution in different populations worldwide. Examination of KIR and ethnic populations may permit analysis of the evolutionary basis for KIR variation, allowing insight into the role of these receptors in health and disease.

The 55 officially recognized ethnic populations of China, which contribute to about 8% of the overall Chinese population, provide abundant genetic resources for KIR–HLA studies. The ethnic groups living in the south and southwest of China can be traced back to three major ancient groups: Di-Qiang, Bai-Pu, and Bai-Yue [15]. According to historical records, the ancient Di-Qiang tribe migrated from northern to southern China before the Qin dynasty in 206 BC and formed several ethnic populations who spoke the language of Tibeto-Burman, which belongs to the Sino-Tibetan linguistic family [15,16]. The ancient Baipu tribe settled down in the south and southwest of Yunnan Province in China and developed into the major Mon-Khmer speaking populations of the Austo-Asiatic linguistic family. Most of the Mon-Khmer ancient tribes migrated to the Indochina Peninsula by the end of 2000 BC, whereas others remained in the Yunnan Province [15,16]. The ancient Baiyue tribe, which was widely distributed along the southeast coast of China, migrated to Yunnan Province and the northern part of Southeast Asia 2000–3000 years ago and then later on migrated to Northern Thailand and contributed to the ancestral gene pool of the Thais [15]. They formed ethnic populations who spoke a language of Daic, part of the Sino-Tibetan linguistic family [17]. There are several ethnic populations in northwestern China, such as the Tu and Yugu. It has been suggested that the Tu population originated from an ancient Xian-Bei tribe, who constructed the Kingdom of Tuguhun in 400 AD, and most of them live in Qinhai and Gansu Provinces. The Yugu originated from Hui-Hu in 600 AD and live only in Gansu Province now. Both the Tu and Yugu integrated with Mongolian and Han populations during the following centuries; moreover, both speak a language of Mongolian belonging to the Altaic linguistic family [15,17].

In the present study, 11 ethnic populations—Hani, Jinuo, Lisu, and Nu, speaking Tibeto-Burman; Bulang and Wa, speaking Mon-Khmer; Dai, Maonan, and Zhuang, speaking Daic; and Tu and Yugu, speaking Mongolian—were selected for HLA and KIR genotyping. The KIR gene’s distribution in Bulang, Nu, Yugu, and Zhuang has been reported previously [18]. The presence/absence of the 16 KIR loci were detected and the KIR gene’s phenotype, genotype, and A and B haplotype frequencies, as well as the KIR ligand’s HLA allotype and KIR–HLA pairs, are reported. Principal component analysis (PCA) and phylogenetic trees were constructed to compare the characteristics of the KIR and KIR–HLA pair distributions in the 11 populations.

## 2. Material and Methods

### 2.1. Subject and Samples

**Figure 1:**
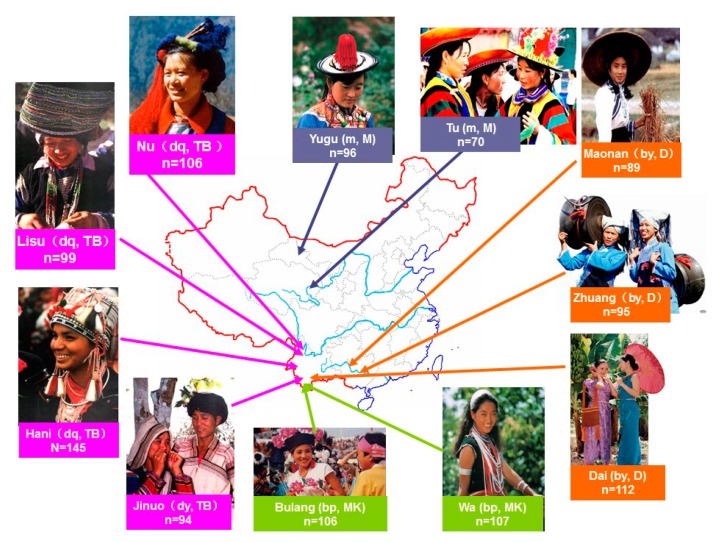
The geographic locations of the 11 ethnic populations in China. dq: Di-Qiang ancient group; bp: Baipu ancient group; by: Baiyue ancient group; m: Mongolian group; TB: Tibeto-Burman sublanguage family; MK: Mon-Khmer sublanguage family; D: Daic sublanguage family; M: Mongolian sublanguage family.

A total of 1119 unrelated individuals were recruited from 11 Chinese ethnic populations in China. The geographic location, sample size of each population, the language family to which they belong, and the original ancient groups they are from are listed in Figure 1 and Supplementary Table S1. These populations are descended from four ancient Chinese groups and belong to four different language subfamilies as mentioned in the introduction. The geographic origin, nationalities, and pedigree (unrelated through at least three generations) of each individual were ascertained before sampling. The present study has been approved by the Committee on the Ethics of Institute of Medical Biology, Chinese Academy of Medical Sciences, the batch number is YIKESHENGLUNZI [2012]12. All the individuals are healthy and gave written informed consent in accordance with the Declaration of Helsinki.

Genomic DNA was extracted from peripheral lymphocytes using a QIAamp Blood Kit (Qiagen, Hilden, Germany), in accordance with the manufacturer’s protocol. DNA samples were quantified with a NanoDrop ND-1000 spectrophotometer (NanoDrop Technologies, Wilmington, WI, USA) and adjusted to a concentration of 20 ng/μL.

### 2.2. KIR Genotyping

The 16 KIR genes were genotyped using the Luminex MultiAnalyte Profiling System (xMAP) with a One Lambda KIR typing kit (One Lambda, Canoga Park, CA, USA), as previously reported [18]. Briefly, three separate PCR products were amplified: exon 3, exon 5, and exons 7–9. The PCR products were run on 2% agarose gel to confirm the specificity and efficiency of the reactions. Then, the PCR amplicons were denatured and hybridized with complementary 81-nucleotide oligonucleotide probes that had been immobilized on fluorescent-coated microsphere beads. At the same time, the biotinylated PCR products were labeled with phycoerythrin-conjugated streptavidin and immediately examined with the Luminex 200 system (Luminex, Austin, TX, USA). Genotype determination and data analysis were performed automatically using the LABScan 100 platform (One Lambda, Canoga Park, CA, USA) in accordance with the manufacturer’s instructions.

### 2.3. Statistical Analysis

Hardy–Weinberg’s equilibrium for each of the alleles was assessed using the Guo and Thompson method [19]. For KIR genes, the observed frequency for each KIR gene was determined via direct counting and corresponded to the ratio of the number within the population that carried the gene to the total population number. KIR locus gene frequencies (KLFs) were estimated by using the formula KLF = 1 − $\sqrt{1-f}$, where *f* is the observed frequency of a particular KIR sequence in a population. The genotypes were defined by referring to the Allele Frequencies website (http://www.allelefrequencies.net). Each genotype was named in accordance with the genotype number. The genotypes that could not be found in the database were named as unknown. Group A and B haplotypes and frequencies were predicted on the basis of a previous study [20].

HLA genotyping of 11 ethnic populations has been reported previously [21,22,23,24,25], and the allelic frequencies are summarized in Supplementary Table S2. The frequencies of *HLA-C1/C2* allotype, *HLA-Bw4* (*Bw4-80I* and *Bw4-80T*)/*Bw6* allotype, and *HLA-A11, -A3* were calculated using direct counting. The *HLA-C1* and *HLA-C2*, *HLA-A11* and *HLA-A3*, and *HLA-Bw4/Bw6* groups were used to analyze KIR–HLA combinations. The observed frequencies of KIR–HLA matched pairs were calculated using direct counting. The significance of the correlations of KIR and HLA frequencies among populations were estimated using the correlation coefficient (r) and the *t*-test was used to establish whether the correlation coefficient was significant using SPSS 16.0 [26]. The chord distance of Nei (Da distances among the populations were calculated based on 11 KIR gene (*2DL1*, *2DL2*, *2DL3*, *3DL1*, *2DS1*, *2DS2*, *2DS3*, *2DS4*, *2DS5*, *3DS1*, and *2DL5*) frequencies; *HLA-A*, *HLA-B*, and *HLA-C* allele frequencies; or HLA–KIR combinations. A neighbor-joining (NJ) tree was constructed using Mega 7.0 software based on the DA distance [27]. Principal component analysis (PCA) was also performed based either on KIR genes, HLA alleles, or KIR–HLA combination frequencies using SPSS 16.0 software [26]. Significant differences in KIR and KIR–HLA pair frequencies between two populations were determined using a contingency test. The difference between the northern and southern groups were detected using a *t*-test with SPSS 16.0 software [26]. A value of *p* < 0.05 was considered to be statistically significant. The observed 11 KIR gene (*2DL1*, *2DL2*, *2DL3*, *3DL1*, *2DS1*, *2DS2*, *2DS3*, *2DS4*, *2DS5*, *3DS1*, and *2DL5*) frequencies of 47 other populations were from previous studies and the DA distance among 58 populations were calculated (Supplementary Tables S5 and S6). The phylogenetic tree was constructed based on the DA distance using the minimum evolution method from Mega 7.0 software [28].

## 3. Results

### 3.1. KIR Gene, Genotype, and Haplotype Frequencies

The observed KIR frequencies and the estimated gene frequencies for each locus in the 11 populations are listed in Table 1. The four framework loci (*KIR3DL3*, *3DP1*, *2DL4*, and *3DL2*) were exhibited in all individuals in 10 populations, except one individual in Yugu, for whom *2DL4* and *3DP1* were not observed. The non-framework pseudogene *2DP1* was observed in all individuals in Hani, Nu, Dai, Zhuang, and Tu, but not in all other populations, with frequencies of 94.8%–99.1%. The frequencies of *3DL1*, *2DL1*, and *2DS4* were about 90%–100% in 11 populations, with the exception in Jinuo and Bulang, which showed frequencies of 88% and 81% at *3DL1* and 88% and 79% at *2DS4*, respectively. Other activating KIRs, including *3DS1*, *2DS1*, *2DS3*, and *2DS5*, as well as inhibitory KIRs, including *2DL2* and *2DL5*, exhibited diverse distributions in different populations. Bulang was different compared with the other 10 populations at *3DS1* and *2DS1* (*p* < 0.05), 9 other populations except Zhuang at *2DS3*, and 9 other populations except Jinuo at *2DS4*. The following difference was among Nu and others. For all populations, the most diverse was at *2DS3* (Supplementary Table S3).

In total, 92 KIR genotypes were identified, including 6 new genotypes: 3 in Tu, 2 in Jinuo, and 1 in Wa (Table 2). Genotypes 1, 2, 4, and 8 were observed in all the populations but showed diverse frequencies. Genotype 1 was predominant in all populations except in Bulang. On the contrary, the predominant genotypes in Bulang were genotype 8 followed by genotypes 1, 2, and 75, as previously reported [18]. Originating from the same Baipu ancient group, Wa did not show a distribution similar to Bulang, with genotype 1 being the most predominant, followed by genotypes 2 and 4. The frequencies of genotype 1 were as high as 0.679 in Nu, 0.510 in Hani, 0.469 in Yugu, 0.465 in Lisu, 0.432 in Zhuang, and 0.425 in Wa.

**Figure 2:**
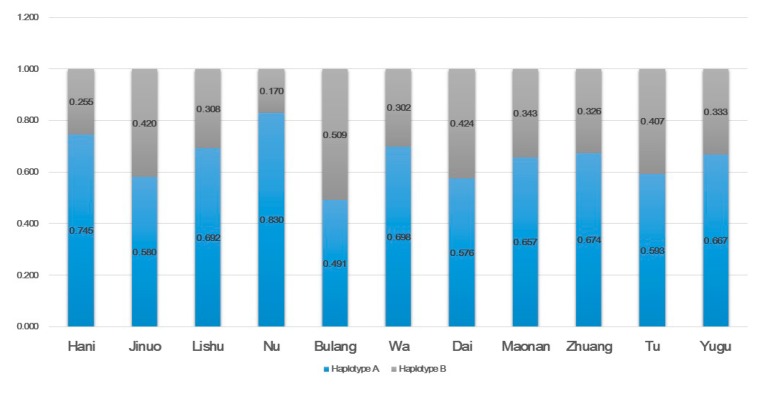
Haplotypes A and B frequencies in 11 ethnic populations in China.

The frequencies of the group A and B haplotypes in the 11 populations were deduced from the genotype data (Figure 2). As with most populations worldwide, the A haplotype was predominant. The frequencies of the A haplotype were around 0.657–0.830 in Nu, Hani, Wa, Lisu, Zhuang, Maonan, and Yugu, while they were around 0.576–0.593 in Tu, Jinuo, and Dai. In Bulang, the frequencies of the A and B haplotypes were almost equal (0.491 vs. 0.509). Haplotype differences were identified among Nu and 10 other populations except Jinuo; among Bulang and Hai, Lisu, Nu, Wa, Maonan, Zhuang, and Yugu; and between Hani and Jinuo, and Dai and Tu (Supplementary Table S3). The distributions of KIR genes, genotypes, and haplotypes did not show any consistency among their original ancient group or linguistic subfamily.

### 3.2. HLA Allotype Frequencies

The frequencies of *HLA-A11/A3, HLA-Bw4* (*Bw4-80I* and *Bw4-80T*), and *HLA-C1* and *HLA-C2* were calculated from the *HLA-A*, *HLA-B*, and *HLA-C* allele genotyping results (Table 3). *HLA-A11/A3* were predominant in all the populations living in southern China, with frequencies higher than 0.556, and they accounted for around 80% of HLA-A alleles in Bulang, Wa, and Hani. On the contrary, *HLA-A11/A3* were around 40% in Tu and Yugu living in northern China. *HLA-Bw4* existed commonly in Tu and Yugu, with frequencies of 0.700 and 0.688, but only with frequencies of 0.283 in Bulang. *HLA-C1* was observed in all individuals in Jinuo, Lisu, Maonan, and Zhuang, with frequencies around >95% in other southern Chinese populations, but with frequencies of 0.914 and 0.833 in Tu and Yugu, respectively, from northern China. On the contrary, the frequencies of *HLA-C2* were around 50% in Tu and Yugu but was only 0.073 in Maonan. This HLA characteristic reflected the northern and southern Chinese original difference, which has been confirmed in previous studies. Therefore, we divided the present study populations into two groups: the southern group, which included Hani, Jinuo, Lisu, Nu, Bulang, Wa, Dai, Maonan, and Zhuang, and the northern group, which included Tu and Yugu. Differences between the northern and southern groups were observed (data not shown).

### 3.3. KIR–HLA Combination

The frequencies of *KIR3DL2* and *HLA-A11/A3* were calculated first. Since the interaction of *KIR2DS4* and *KIR2DS2* with *HLA-A11* has been demonstrated, their combinations have also been calculated [14,29]. The frequencies of *KIR3DL2+A11/A3* and *KIR2DS4+A11/A3* were from 0.417 to 0.879, while the frequencies of *KIR2DS2+A11* were lower, with frequencies from 0.038 to 0.299 (Table 4). In Nu, the frequency of *KIR2DS2+A11* was only 0.038, though HLA-A*11:01 was predominant, with a frequency of 0.411(Table 4).

The individuals carrying either *KIR3DL1* or *KIR3DS1* and its *HLA-Bw4* ligands, or carrying both *KIR3DL1* and *KIR3DS1* together with its HLA-Bw4 ligands, were counted for the KIR–HLA combination (Table 5). *KIR3DL1/3DS1+Bw4* was commonly exhibited in all populations, with frequencies of 0.383–0.700, except in Bulang. The total frequency of the *KIR3DL1/3DS1+Bw4* combination was 0.283, and the frequency of either *KIR3DL1+Bw4* or *KIR3DS1+Bw4* pairs was 0.057. The frequencies of *3DL1+3DS1+Bw4* were predominant in Bulang, and *3DL1+3DS1+Bw4* and *3DL1+Bw4* were almost similar in Jinuo; however, *KIR3DL1+Bw4* was predominant in other 9 populations. In Hani and Wa, all individuals carried either *3DL1+Bw4* or *3DL1+3DS1+Bw4* together, and no one carrying only *3DS1+Bw4* was observed.

Further analysis of KIR with the presence of isoleucine at position 80(*Bw4-80I*) as well as with the presence of threonine at position 80(*Bw4-80T*) was performed. One individual in Dai and Jinuo with *HLA-Bw4* were unable to have their KIR ligands identified, two individuals in Lisu were not able to have their KIR ligands identified, while all the other individuals with *KIR-Bw4* ligands were identified. For the *3DL1/3DS1+Bw4* pair, the frequencies of *80I3DL1+Bw4 80I* were higher than *3DS1+Bw4 80I*. Moreover, in Hani, Nu, Wa, and Maonan, no individuals who only carried *3DS1+Bw4 80I* were observed. There were more individuals only carrying *KIR3DL1+Bw4-80I* than those only carrying *KIR3DL1+Bw4-80T* in Hani, Lisu, Bulang, and Yugu, while there were fewer in Jinuo, Dai, and Maonan, and there was no difference in Nu, Wa, Zhuang, and Tu. There was a similar finding for the *KIR3DL1+Bw4-80T* and *KIR3DS1+Bw4-80T* pairs. Further analysis of the southern and northern groups indicated that the frequencies of *KIR3DL1+Bw4* were lower in the southern group than in the northern group (0.451 ± 0.120 vs. 0.659 ± 0.018, *p* = 0.001).

*KIR2DL2/2DL3* and its ligand *HLA-C1* specifically control NK cell response. Its combination commonly exists in all the populations, but the frequencies were higher in the southern group (0.978 ± 0.006, 95% CI: 0.966–0.988) than in the northern group (0.860 ± 0.020, 95% CI: 0.833–0.886) (*p* = 0.00005). *KIR2DL1* and *KIR2DS2* had a similar binding specificity for *HLA-C2*. The frequencies of *2DL1+HLA-C2* were lower in the southern group (0.211 ± 0.026, 95% CI: 0.162–0.264) than in the northern group (0.472 ± 0.021, 95% CI: 0.443–0.500) (*p* = 0.003). However, there was no difference in frequencies of *2DS1+HLA-C2* combinations between the southern and northern groups (Table 6).

The comparison between the two populations indicated that Yugu and Tu did not show any difference in any of the KIR–HLA pairs, while the other populations all showed differences of between 1 and 15 pairs (Supplementary Table S4). The *2DL2/3+HLA-C1, 2DL1+HLA-C2*, and *2DS1+HLA-C2* pairs showed clear differences between the northern and southern groups. For other *HLA-Bw* or *HLA-A11/A3* pairs, the difference between the two populations was extensive. For example, Wa showed differences with: every other population except Lisu and Nu for carrying both *KIR3DL1+Bw4* and *KIR3DS1+Bw4* pairs; every other population except Jinuo, Nu, and Maonan for carrying both *KIR3DL1+Bw4-80I* and *KIR3DS1+Bw4-80I* pairs; and Hani, Lisu, Nu, Bulang, Dai, and Yugu for carrying *KIR3DL1+Bw4-80T* and *KIR3DS1+Bw4-80T*. For carrying *KIR3DL1+Bw4*, Bulang showed differences with every population except Jinuo, and Jinuo, Wa, Zhuang, Tu, and Yugu showed differences with at least five populations. The differences at *KIR3DL1+Bw4-80I* and *KIR3DL1+Bw4-80T* between the populations were not same as for *KIR3DL1+Bw4*. The differences in carrying *KIR3DL2+A11/A3* or *KIR2DS4+A11/A3* among the populations were similar, and Hani, Bulang, Wa, Tu, and Yugu showed differences with at least five populations.

### 3.4. HLA/KIR Correlation

**Figure 3:**
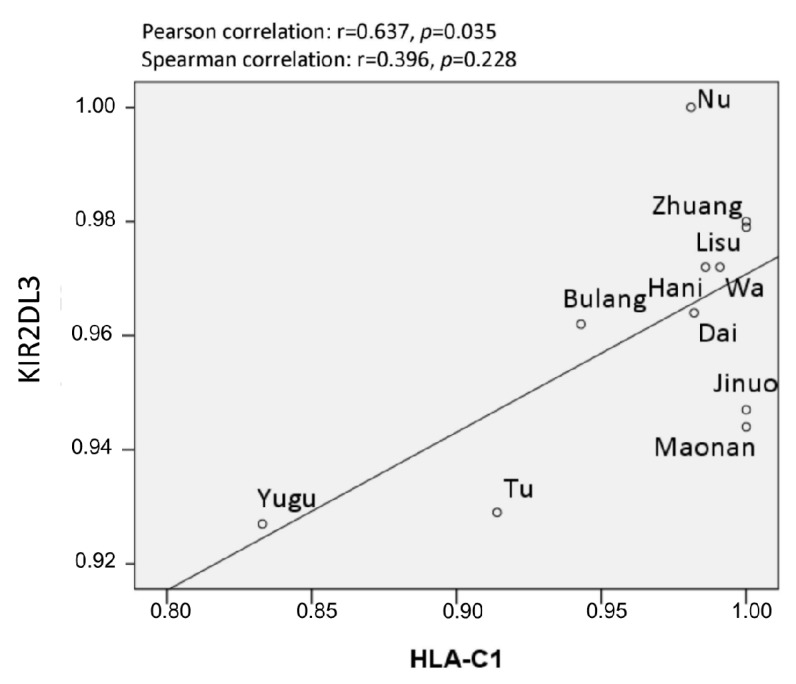
Correlation of KIR2DL3 and HLA-C1.

The correlations of each KIR–HLA receptor and ligand pair were analyzed. The observed frequencies for *KIR2DL3* and *HLA-C1* ligands showed a significant correlation (r = 0.637, *p* = 0.035). Positive correlations were also observed in *KIR3DL1+HLA-Bw4, KIR3DL1+HLA-Bw4 80I, KIR3DL1+HLA-Bw4 80T, KIR2DL2+HLA-C1, KIR2DS1+HLA-C2*, and *KIR2DS2+HLA-C1* pairs, but they were not significant. Negative correlations between *KIR3DS1* and *HLA-Bw4*, and *KIR2DL1* and *HLA-C2* were observed, but they were not significant neither (Figure 3 and Supplementary Figure S1).

### 3.5. Phylogenetic Analysis

**Figure 4:**
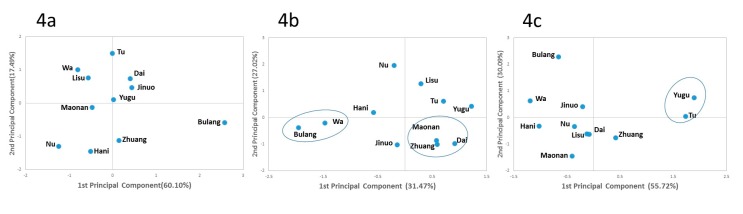
Principal component analysis. (**a**) PCA based on 11 KIR genes. Contributions of the first and second components were 60.10% and 17.49%, respectively. (**b**) PCA based on *HLA-A*, *-B*, and *-C* allele frequencies. Contributions of first and second components were 31.47% and 27.02%, respectively. (**c**) PCA based on KIR–HLA pairs. Contributions of first and second components were 55.72% and 30.09%, respectively.

Both principal component analysis (PCA) and phylogenetic trees using KIR, HLA, and KIR–HLA combination frequencies were employed. For PCA based on 11 KIR (*3DL1*, *2DL1*, *2DL3*, *2DS4*, *2DL2*, *2DL5*, *3DS1*, *2DS1*, *2DS2*, *2DS3*, and *2DS5*) plots, Bulang showed distance from the other 10 populations (Figure 4a), and the other 10 populations did not cluster with their linguistic family as based on *HLA-A*, *-B*, and *-C* (Figure 4b), which agreed with previous studies. On the PCA plots based on KIR–HLA pairs, Yugu and Nu from northern China clustered together and showed distance from the other nine populations from southern China (Figure 4c).

**Figure 5:**
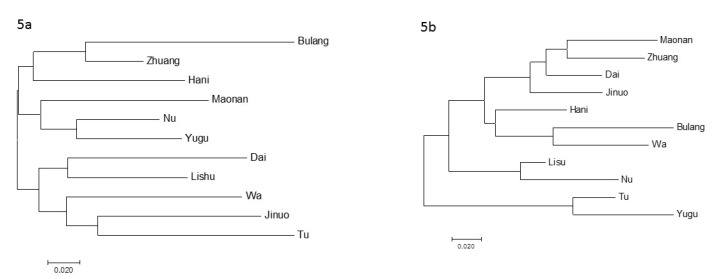
Neighbor-joining tree. (**a**) Neighbor-joining tree based on DA genetic distance from 11 KIR gene frequencies. The optimal tree was one with the sum of branch length = 1.166. (**b**) Neighbor-joining tree based on DA genetic distance from *HLA-A*, *-B*, and *-C* allele frequencies. The optimal tree was one with the sum of branch length = 0.875.

**Figure 6:**
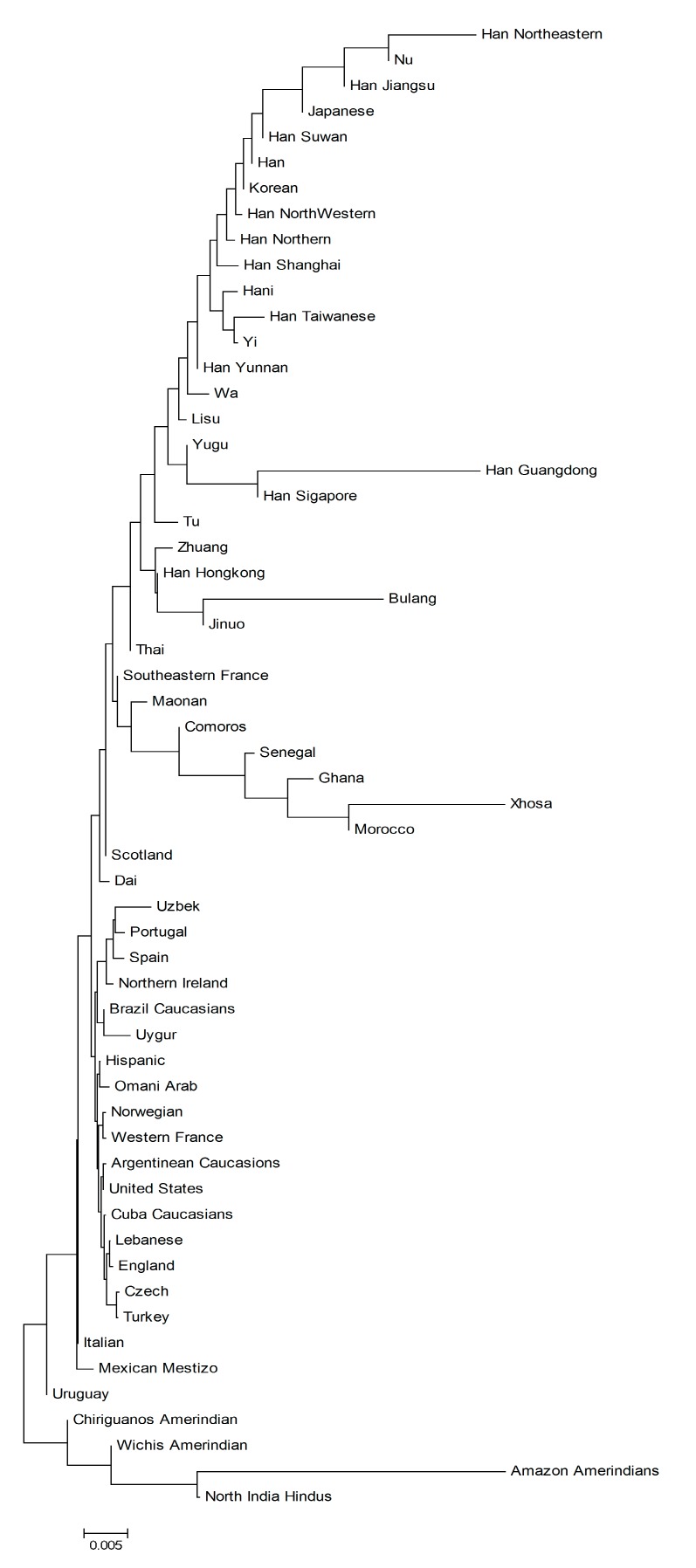
Neighbor-joining tree constructed using 11 KIR genes frequencies of 58 populations worldwide. The optimal tree was one with the sum of branch length = 0.159.

On the phylogenetic tree based on KIRs, for the trees constructed either by all 11 KIR genes (Figure 5a), or by the inhibitor KIR genes or by the activating KIR genes (data not shown) had no clear clustering among the populations. For the NJ tree constructed using HLA genes, Tu and Yugu clustered together as one major branch. Bulang and Wa of Khmer clustered together, and Maonao, Zhuang, and Dai of Daic clustered together with Jinuo (Figure 5b). When 58 populations were compared on the phylogenetic tree using 11 KIRs frequencies, most populations clustered together according to their geographic location of Asian, European, African, and American (Figure 6). However, the closeness were not displayed clearly as in the NJ tree constructed by the HLA genes, in which the populations clustered according to their evolutional relationship [25]. In the Asian branch, on one hand, most Han populations in northern China clustered with Japanese and Korean, but also together with Nu, Yi, and Hani ethnic populations living in southern China. On the other hand, Yugu and Tu living in Northern China clustered with Southern Hans living in Guangdong, Hong Kong, together with Maonan, Bulang, Jinuo, etc., ethnic populations in southern China. The 11 ethnic populations in the present study still did not show a clear origin or linguistic clustering trend.

## 4. Discussion

The extensive diversity of HLA and KIR genes and their interactive roles in the immune response make these genes coevolve in genotypic combination. Disease and population studies have confirmed that they evolved together as specific KIR–HLA pairs to regulate NK cell function and play a vital role in the innate defense against pathogens and early placentation [30,31,32]. KIR–HLA combination studies have been performed in different populations worldwide; however, previous studies in China were limited to the Han population [4,33,34,35,36,37,38]. In the present study, we analyzed KIR and its HLA pairs in 11 ethnic populations from northern and southern China covering four different linguistic language families that represent the major origins of Chinese ethnic populations. This study not only provides useful genomic diversity data for the future study of viral infections, autoimmune diseases, and reproductive fitness among these populations, but also reveals clues about HLA and KIR interaction and coevolution under the diverse change of pathogen infections.

The coevolution of HLA and KIR has been proved by several population studies worldwide, and different KIR–HLA pair correlations were identified in different populations. In 2006, Single et al. studied the distribution of KIR and its HLA ligands in 30 populations worldwide and observed that a balancing selection acted on the negative correlation between *KIR3DS1* and *HLA-Bw4-80I* pairs [30]. In 2013, Hollenbach et al. compared KIR–HLA pairs in 105 populations worldwide and revealed a significant correlation between *KIR2DL3* and *HLA-C* ligands. However, the correlation for *KIR3DL1* and *HLA-Bw4* pairs was not significant [39]. In the Italian population, a correlation between *KIR* and *HLA-C* ligands was not observed; instead, a correlation between *KIR3DL1* and *HLA-Bw4* ligands, as well as *KIR3DL2* and *HLA-A3* and *HLA-A11* ligands, was observed [40]. In the present study, the observed frequencies for *KIR2DL3* and *HLA-C1* ligands showed a correlation (r = 0.637, *p* = 0.035) that agreed with Hollenbach et al.’s study, as well as Gendzekhadze’s study in the Yucpa og South Amerindian [4]. The association of *KIR2DL3* and *HLA-C1* has also been investigated regarding the Hepatitis C virus and Malaria infection [41,42]. In Hirayasu et al.’s study, they found *KIR2DL3+HLA-C1*, but no other KIR–HLA pairs were associated with cerebral malaria, and the frequency of combination was significant lower in malaria high-endemic populations. This result suggested that natural selection has reduced the *KIR2DL3+HLA-C1* frequencies in malaria high-endemic populations to favor the development of malaria [41]. Thus, KIR–HLA coevolution may be driven by microbial pathogens, resulting in specific distributions of KIR–HLA pairs in different populations.

The genetic difference between southern and northern Chinese has been confirmed in studies of HLA [35,43], as well as immunoglobulins [44], microsatellites [45], and Y-chromosome single-nucleotide polymorphisms [46]. Furthermore, population migration from northern to southern China has frequently happened throughout Chinese history [15,16,17]. In the present study, *KIR2DL2/3+HLA-C1*, *KIR2DL1+HLA-C2*, and *KIR2DS1+HLA-C2* pairs showed clear differences between the northern and southern groups. The frequencies of *KIR2DL2/3+HLA-C1* pairs were significantly higher in the southern group than in the northern group (0.978 vs. 0.860, *p* = 0.00005). This difference has also been investigated in Han population. The frequencies of *KIR2DL2/3+HLA-C1* pairs were higher in two southern Chinese Han, namely Guangdong Han and Yunnan Han, than in the northern ethnic group, with frequencies of 0.981 and 0.950, respectively. In contrast, the frequencies of *KIR2DL1+HLA-C2* and *KIR2DS1+HLA-C2* pairs were lower than in the northern group, with frequencies of 0.294 and 0.351 for *KIR2DL1+HLA-C2*, and 0.100 and 0.155 for *KIR2DS1+HLA-C2* in Guangdong Han and Yunnan Han, respectively [36,38]. Furthermore, the Tu and Yugu formed a cluster and showed a distance from other ethnic populations in southern China in the PCA plot constructed using KIR–HLA pair frequencies. According to historical records, northern and southern China underwent different pathogenic pressures. Regarding malaria (a serious infectious disease prevalent in China since 2700 BC), its epidemic area was focused in southern China, whereas northern China was malaria free or had a very low incidence rate [47,48]. Historically, Yunnan Province has been the most high-risk malaria area, especially along the China–Myanmar border [49,50]. In the present study, Tu and Yugu are from northern China, while the other populations are all in southern China, the most high-risk malaria areas. Therefore, we deduced that the different distribution between the northern and southern groups in China may have been caused by severe infectious disease epidemics, such as malaria.

Except for *KIR+HLA-C* pairs, *KIR+HLA-Bw* and *KIR+HLA-A3/A11* pair differences were diverse in different populations. When considering KIR genes, genotypes, and haplotypes, more diversity was exhibited. It is interesting to note that the HLA gene distributions were in accordance with the population linguistic group and their origins. Both in the PCA plot and neighbor-joining tree constructed using HLA allele frequencies, the same linguistic origin populations clustered together. However, there were no clear cluster trends to distinguish the populations according to their origin or linguistic classification using either KIR, activated KIR, or KIR genotype frequencies among 11 ethnic populations. Moreover, in the phylogenetic tree constructed using 11 KIRs frequencies of 58 populations worldwide, there was a geographic closeness among Asians, Africans, Europeans, and Americans, while the 11 ethnic populations in the present study still did not show a clear origin or linguistic clustering trend. Compared with HLA genes, KIR genes have experienced a rapid evolution through a combination of gene duplication and nonhomologous recombination [1]. The extensive diversity of KIR genes in different populations worldwide indicates that distinct diseases have recently acted or are still acting to select on KIR repertoires [2]. This evolution was thought to be driven by the selective pressure of pathogen invasion, as well as reproduction. Moreover, haplotypes A and B are thought to have maintained a balance selection in human beings. The A haplotypes are associated with an improved response to pathogens, while B haplotypes are associated with reproductive fitness [51,52]. Previous studies have indicated that the populations are related to their geographic distribution based on KIR haplotype B but do not show a correlation based on haplotype A [53]. Moreover, it has been reported that B haplotypes are more prevalent in Australian Aborigines and Asian Indians, where the possible reason is due to these populations maybe being under strong pressure from infectious diseases [2]. In the present study, the frequencies of haplotypes A and B were almost similar to each other in Bulang, which showed a difference from other Asian populations. On the contrary, in Nu, haplotype A was as high as 0.830, which showed a significant difference from the other 10 populations. This extensive range of haplotype A in the present study, from 0.491 in Bulang to 0.830 in Nu, together with the diverse frequencies worldwide, may be the result of a founder effect, genetic drift, or natural selection [5,31]. Therefore, the distribution of KIR profiles among the present study populations could not be interpreted as a phylogenetic tree.

In conclusion, the distribution of KIR and its HLA ligands in 11 ethnic populations in China exhibited diverse characteristics, where each group had its specific KIR and KIR–HLA pair profile. The difference of KIR–HLA pairs between the northern and southern groups, but not among the four original groups, may reflect the strong pressure from previous or ongoing infectious diseases that have had a significant impact on KIR and its HLA combination repertoires.

## Figures and Tables

**Table 1:** The observed KIR frequencies and the estimated gene frequencies for each locus in 11 ethnic populations.

	Hani (n = 145)		Jinuo (n = 94)		Lisu (n = 99)		Nu (n = 106)		Bulang (n = 106)		Wa (n = 107)		Dai (n = 112)		Maonan (n = 89)		Zhuang (n = 95)		Tu (n = 70)		Yugu (n = 96)	
	OF (%)	GF	OF (%)	GF	OF (%)	GF	OF (%)	GF	OF (%)	GF	OF (%)	GF	OF (%)	GF	OF (%)	GF	OF (%)	GF	OF (%)	GF	OF (%)	GF
*3DL1*	99	0.917	88	0.658	92	0.716	98	0.863	81	0.566	98	0.863	91	0.701	99	0.894	94	0.749	96	0.793	94	0.750
*2DL1*	100	1.000	100	1.000	99	0.899	100	1.000	98	0.863	100	1.000	97	0.836	99	0.894	100	1.000	100	1.000	95	0.772
*2DL3*	97	0.834	95	0.769	98	0.858	100	1.000	96	0.806	97	0.832	96	0.811	94	0.763	98	0.855	93	0.733	93	0.730
*2DS4*	99	0.917	88	0.658	92	0.716	98	0.863	79	0.544	98	0.863	90	0.687	99	0.894	94	0.749	97	0.831	92	0.711
*2DL2*	27	0.145	27	0.143	33	0.184	10	0.053	16	0.084	32	0.176	41	0.232	39	0.221	29	0.160	33	0.181	27	0.146
*2DL5*	30	0.165	35	0.194	34	0.190	26	0.142	70	0.451	24	0.126	53	0.312	35	0.193	46	0.267	33	0.181	45	0.257
*3DS1*	34	0.186	50	0.293	30	0.165	25	0.131	73	0.477	31	0.170	41	0.232	35	0.193	41	0.232	53	0.313	35	0.196
*2DS1*	34	0.191	44	0.249	38	0.215	23	0.120	73	0.477	34	0.187	43	0.244	30	0.165	37	0.205	39	0.216	41	0.229
*2DS2*	27	0.145	27	0.143	33	0.184	10	0.053	16	0.084	32	0.176	41	0.232	39	0.221	29	0.160	33	0.181	27	0.146
*2DS3*	34	0.191	29	0.156	8	0.041	8	0.038	48	0.280	11	0.058	28	0.150	31	0.172	37	0.205	13	0.066	21	0.110
*2DS5*	9	0.046	39	0.221	29	0.159	16	0.084	41	0.229	37	0.205	33	0.182	20	0.107	15	0.077	43	0.244	31	0.171
*2DL4*	100	1.000	100	1.000	100	1.000	100	1.000	100	1.000	100	1.000	100	1.000	100	1.000	100	1.000	100	1.000	99	0.898
*3DL2*	100	1.000	100	1.000	100	1.000	100	1.000	100	1.000	100	1.000	100	1.000	100	1.000	100	1.000	100	1.000	100	1.000
*3DL3*	100	1.000	100	1.000	100	1.000	100	1.000	100	1.000	100	1.000	100	1.000	100	1.000	100	1.000	100	1.000	100	1.000
*2DP1*	100	1.000	99	0.897	99	0.899	100	1.000	98	0.863	99	0.903	100	1.000	99	0.894	100	1.000	100	1.000	95	0.772
*3DP1*	100	1.000	100	1.000	100	1.000	100	1.000	100	1.000	100	1.000	100	1.000	100	1.000	100	1.000	100	1.000	99	0.898

OF: Observed Frequencies, GF: estimated Gene frequencies.

**Table 2:** KIR genotypes identified in 11 ethnic populations.

Hapl Group	Geno Group	Genotype ID	*3DL1*	*2DL1*	*2DL3*	*2DS4*	*2DL2*	*2DL5*	*3DS1*	*2DS1*	*2DS2*	*2DS3*	*2DS5*	*2DL4*	*3DL2*	*3DL3*	*2DP1*	*3DP1*	Hani	Jinuo	Lisu	Nu	Bulang	Wa	Dai	Maonan	Zhuang	Tu	Yugu
																			Fre.	Fre.	Fre.	Fre.	Fre.	Fre.	Fre.	Fre.	Fre.	Fre.	Fre.
AA	AA	1	1	1	1	1								1	1	1	1	1	0.510	0.287	0.465	0.679	0.208	0.425	0.286	0.393	0.432	0.271	0.469
AA	AA	180	1	1		1								1	1	1	1	1	0.007	0.021	0.010							0.014	
AA	AA	203	1	1	1	1								1	1	1		1						0.009					
AA	AA	332	1		1	1								1	1	1	1	1							0.009				
Bx	AB	2	1	1	1	1		1	1	1			1	1	1	1	1	1	0.034	0.117	0.101	0.113	0.170	0.113	0.080	0.056	0.074	0.143	0.094
Bx	AB	3	1	1	1	1	1	1	1	1	1		1	1	1	1	1	1	0.007	0.021	0.040	0.009	0.028	0.009	0.063		0.021	0.043	0.021
Bx	AB	4	1	1	1	1	1				1			1	1	1	1	1	0.069	0.074	0.091	0.057	0.028	0.085	0.107	0.135	0.095	0.143	0.052
Bx	AB	5	1	1	1	1	1	1			1	1		1	1	1	1	1	0.007	0.011	0.020		0.019	0.019	0.063	0.022	0.042		0.021
Bx	AB	6	1	1	1	1	1	1	1	1	1	1	1	1	1	1	1	1	0.021	0.011		0.009			0.018	0.067	0.011		0.042
Bx	AB	7	1	1	1	1	1	1	1	1	1	1		1	1	1	1	1	0.055	0.011	0.030		0.028	0.009	0.009		0.063	0.014	
Bx	AB	8	1	1	1	1		1	1	1		1		1	1	1	1	1	0.076	0.053	0.010	0.057	0.255	0.019	0.063	0.101	0.137	0.057	0.083
Bx	AB	9	1	1	1	1	1	1		1	1		1	1	1	1	1	1	0.007		0.030	0.009		0.038	0.009	0.011			0.042
Bx	AB	11	1	1	1	1	1	1		1	1	1		1	1	1	1	1	0.021			0.009			0.018				
Bx	AB	12	1	1	1	1		1	1	1	1		1	1	1	1	1	1							0.009				
Bx	AB	13	1	1	1	1	1	1	1		1	1		1	1	1	1	1	0.034		0.010				0.009	0.022	0.032	0.014	
Bx	AB	14	1	1	1	1			1					1	1	1	1	1		0.032	0.020				0.018	0.022	0.011	0.014	
Bx	AB	15	1	1	1	1				1				1	1	1	1	1	0.007		0.010				0.009				
Bx	AB	17	1	1	1	1		1	1				1	1	1	1	1	1										0.014	
Bx	AB	19	1	1	1	1	1							1	1	1	1	1							0.009				
Bx	AB	23	1	1	1	1							1	1	1	1	1	1		0.011				0.019	0.009			0.014	
Bx	AB	25	1	1	1	1	1	1			1	1	1	1	1	1	1	1							0.009				
Bx	AB	27	1	1	1	1		1	1			1		1	1	1	1	1							0.009				
Bx	AB	30	1	1	1	1		1				1		1	1	1	1	1					0.009		0.009	0.011			0.010
Bx	AB	31	1	1	1	1	1	1			1			1	1	1	1	1		0.021									
Bx	AB	33	1	1	1	1		1	1	1				1	1	1	1	1	0.007										
Bx	AB	35	1	1	1	1		1		1			1	1	1	1	1	1		0.011				0.009	0.009				
Bx	AB	41	1	1	1	1	1		1		1	1		1	1	1	1	1		0.011						0.011			
Bx	AB	44	1	1	1	1	1				1		1	1	1	1	1	1	0.007					0.038		0.011			
Bx	AB	57	1	1	1	1		1	1	1	1	1		1	1	1	1	1							0.009				
Bx	AB	62	1	1	1	1	1				1	1		1	1	1	1	1		0.021						0.011			
Bx	AB	63	1	1	1	1	1		1	1	1		1	1	1	1	1	1		0.011				0.028					
Bx	BB	68		1	1		1	1	1	1	1		1	1	1	1	1	1			0.030	0.009	0.009		0.009	0.011			
Bx	BB	69		1	1			1	1	1			1	1	1	1	1	1		0.011	0.020	0.009	0.038	0.009	0.027				0.031
Bx	BB	70		1	1		1	1	1	1	1	1	1	1	1	1	1	1		0.011	0.010		0.009		0.018		0.011		
Bx	BB	71	1	1		1	1	1			1	1		1	1	1	1	1	0.014	0.011						0.011	0.021		0.010
Bx	BB	72	1			1	1				1			1	1	1		1					0.009						0.010
Bx	BB	73	1	1		1	1	1	1	1	1	1	1	1	1	1	1	1							0.018				
Bx	BB	75		1	1			1	1	1		1	1	1	1	1	1	1	0.007	0.053			0.104				0.032		0.010
Bx	BB	76	1			1	1	1	1	1	1		1	1	1	1		1											0.021
Bx	BB	79	1	1	1			1	1	1			1	1	1	1	1	1											0.010
Bx	BB	80	1	1	1		1	1	1	1	1		1	1	1	1	1	1					0.009						0.010
Bx	BB	81		1			1	1	1	1	1	1	1	1	1	1	1	1											0.010
Bx	BB	86		1	1	1	1	1	1	1	1		1	1	1	1	1	1										0.014	
Bx	BB	89	1	1		1	1				1			1	1	1	1	1								0.022			
Bx	BB	97	1			1	1	1		1	1	1		1	1	1		1								0.011			0.010
Bx	BB	104	1			1	1				1			1	1	1	1	1							0.009				
Bx	BB	106	1			1	1	1		1	1		1	1	1	1		1			0.010								
Bx	BB	113	1	1		1	1	1		1	1	1		1	1	1	1	1	0.007					0.009					
Bx	BB	117		1	1			1	1	1		1		1	1	1	1	1		0.011			0.019		0.009		0.021		
Bx	BB	151		1	1	1	1	1	1	1	1	1		1	1	1	1	1								0.011			
Bx	BB	154		1	1	1		1	1	1			1	1	1	1	1	1							0.009				
Bx	BB	164		1	1		1	1		1	1		1	1	1	1	1	1			0.010								
Bx	AB	188	1	1	1	1	1		1		1			1	1	1	1	1						0.009					
Bx	AB	192	1	1	1	1	1			1	1			1	1	1	1	1			0.030			0.019					
Bx	BB	194					1	1		1	1		1		1	1													0.010
Bx	AB	202	1	1	1	1			1	1			1	1	1	1	1	1		0.074	0.020		0.019	0.028	0.009	0.011		0.057	
Bx	AB	205	1	1	1	1				1		1		1	1	1	1	1											0.010
Bx	AB	233	1	1	1	1	1		1	1	1	1		1	1	1	1	1	0.014					0.009				0.014	
Bx	BB	243		1			1	1	1	1	1		1	1	1	1	1	1							0.009				
Bx	BB	247	1	1		1		n	1	1		1		1	1	1	n	n					0.009						
Bx	AB	260	1	1	1	1						1		1	1	1	1	1								0.011			
Bx	AB	264	1	1	1	1			1			1		1	1	1	1	1	0.007	0.011				0.019				0.014	
Bx	AB	268	1	1	1	1	1				1	1	1	1	1	1	1	1						0.009					
Bx	BB	280		1	1		1		1	1	1	1	1	1	1	1	1	1		0.011									
Bx	BB	289	1	1		1			1	1			1	1	1	1	1	1						0.009					
Bx	BB	293	1		1	1	1				1			1	1	1	1	1							0.009				
Bx	BB	317	1	1		1	1				1	1		1	1	1	1	1		0.021								0.014	
Bx	AB	319	1	1	1	1	1		1	1	1	1	1	1	1	1	1	1		0.011						0.011			
Bx	BB	320					1	1	1	1	1	1	1	1	1	1		1					0.009						
Bx	BB	325	1	1			1	1	1	1	1	1	1	1	1	1	1	1					0.009						
Bx	BB	331	1	1	1			1	1	1		1		1	1	1	1	1							0.009				
Bx	AB	370	1	1	1	1	1			1	1		1	1	1	1	1	1			0.010			0.009				0.014	
Bx	AB	372	1	1	1	1			1	1		1		1	1	1	1	1	0.076	0.011			0.009	0.009					
Bx	BB	375		1	1					1			1	1	1	1	1	1		0.011									
Bx	BB	379	1	1		1	1		1	1	1		1	1	1	1	1	1										0.014	
Bx	AB	381	1	1	1	1	1	1	1	1	1			1	1	1	1	1			0.010								
Bx	AB	382	1	1	1	1	1	1		1	1	1	1	1	1	1	1	1	0.007						0.009				
Bx	BB	390		1	1			1		1			1	1	1	1	1	1			0.010				0.009				
Bx	BB	394	1	1		1		1	1	1			1	1	1	1	1	1										0.014	
Bx	AB	400	1	1	1	1	1		1		1		1	1	1	1	1	1		0.011				0.019		0.011		0.029	0.010
Bx	BB	402		1	1			1	1				1	1	1	1	1	1										0.014	
Bx	AB	433	1	1	1	1		1	1					1	1	1	1	1				0.038							0.010
Bx	BB	466	1	1		1		1	1	1		1		1	1	1	1	1								0.011			
Bx	AB	570	1	1	1	1	1		1		1	1	1	1	1	1	1	1						0.009					
Bx	BB	578	1	1	1		1	1					1	1	1	1	1	1							0.009				
Bx	AB	587	1	1	1	1	1	1	1		1			1	1	1	1	1							0.009				
Bx	AB	n1*	1	1	1	1			1				1	1	1	1	1	1										0.029	
Bx	AB	n2*	1	1	1	1			1			1	1	1	1	1		1		0.011									
Bx	BB	n3*	1	1		1	1		1		1		1	1	1	1	1	1										0.014	
Bx	BB	n4*		1	1				1				1	1	1	1	1	1										0.014	
Bx	BB	n5*		1	1				1			1	1	1	1	1	1	1		0.011									
Bx	BB	n6*		1					1	1			1	1	1	1	1	1						0.009					

Fre.: Frequency. *n1–n6: unknown genotype ID.

**Table 3:** HLA allotype frequencies in 11 ethnic populations.

Title	Hani (n = 145)	Jinuo (n = 94) *	Lisu (n = 99)	Nu (n = 106)	Bulang (n = 106)	Wa (n = 107)	Dai (n = 112)	Maonan (n = 89)*	Zhuang (n = 95)	Tu (n = 70)	Yugu (n = 96)
*A11/A3*	0.821	0.585	0.556	0.642	0.792	0.879	0.643	0.697	0.579	0.443	0.417
*Bw4*	0.462	0.394	0.505	0.472	0.283	0.393	0.607	0.596	0.653	0.700	0.688
*Bw4-80I*	0.400	0.074	0.394	0.198	0.179	0.196	0.321	0.135	0.400	0.400	0.448
*Bw4-80T*	0.062	0.330	0.162	0.283	0.104	0.215	0.411	0.494	0.305	0.386	0.333
*HLA-C1*	0.986	1.000	1.000	0.981	0.943	0.991	0.982	1.000	1.000	0.914	0.833
*HLA-C2*	0.145	0.258	0.162	0.208	0.406	0.262	0.179	0.073	0.221	0.443	0.542
*C1/C1*	0.855	0.742	0.838	0.792	0.594	0.738	0.821	0.927	0.779	0.557	0.458
*C1/C2*	0.131	0.258	0.162	0.189	0.349	0.252	0.161	0.073	0.221	0.357	0.375
*C2/C2*	0.014	0.000	0.000	0.019	0.057	0.009	0.018	0.000	0.000	0.086	0.167

* The numbers for *HLA-C1* and *HLA-C2* was 89 in Jinuo and was 82 in Maonan.

**Table 4:** Distribution of KIR and HLA-A11/A3 pairs in 11 ethnic populations.

Title	*3DL2*+*A11/A3*		*2DS4*+*A11/A3*		*2DS2*+*A11*	
	Counts	Fre.	Counts	Fre.	Counts	Fre.
Hani (n = 145)	119	0.821	119	0.821	31	0.214
Jinuo (n = 94)	55	0.585	46	0.489	12	0.128
Lisu(n = 99)	55	0.556	50	0.505	21	0.212
Nu (n = 106)	68	0.642	68	0.642	4	0.038
Bulang (n = 106)	84	0.792	65	0.613	15	0.142
Wa (n = 107)	94	0.879	92	0.860	32	0.299
Dai (n = 112)	72	0.643	66	0.589	30	0.268
Maonan (n = 89)	62	0.697	58	0.652	24	0.270
Zhuang (n = 95)	55	0.579	49	0.516	16	0.168
Tu (n = 70)	31	0.443	30	0.429	11	0.157
Yugu (n = 96)	40	0.417	38	0.396	10	0.104

Fre.: Frequency.

**Table 5:** Distributions of KIR and HLA-B pairs in 11 ethnic populations.

	*3DL1*+*3DS1*+*Bw4*		*3DL1*+*Bw4*		*3DS1*+*Bw4*		*3DL1*+*3DS1*+*BW4 80I*		*3DL1*+*BW4 80I*		*3DS1*+*BW4 80I*		*3DL1*+*3DS1*+*BW4 80T*		*3DL1*+*BW4 80T*		*3DS1*+*BW4 80T*	
	Counts	Fre.	Counts	Fre.	Counts	Fre.	Counts	Fre.	Counts	Fre.	Counts	Fre.	Counts	Fre.	Counts	Fre.	Counts	Fre.
Hani (n = 145)	23	0.159	44	0.303	0	0.000	21	0.145	37	0.255	0	0.000	2	0.014	9	0.062	0	0.000
Jinuo (n = 94)	15	0.160	17	0.181	4	0.043	0	0.000	6	0.064	1	0.011	15	0.160	13	0.138	2	0.021
Lisu (n = 99)	13	0.131	32	0.323	4	0.040	12	0.121	24	0.242	2	0.020	2	0.020	11	0.111	2	0.020
Nu (n = 106)	11	0.104	38	0.358	1	0.009	7	0.066	20	0.189	0	0.000	4	0.038	26	0.245	1	0.009
Bulang (n = 106)	18	0.170	6	0.057	6	0.057	10	0.094	5	0.047	3	0.028	5	0.047	3	0.028	3	0.028
Wa (n = 107)	7	0.065	35	0.327	0	0.000	2	0.019	19	0.178	0	0.000	5	0.047	18	0.168	0	0.000
Dai (n = 112)	21	0.188	38	0.339	8	0.071	14	0.125	19	0.170	2	0.018	14	0.125	24	0.214	8	0.071
Maonan (n = 89)	23	0.258	29	0.326	1	0.011	5	0.056	7	0.079	0	0.000	20	0.225	23	0.258	1	0.011
Zhuang (n = 95)	19	0.200	39	0.411	4	0.042	10	0.105	23	0.242	3	0.032	10	0.105	22	0.232	2	0.021
Tu (n = 70)	20	0.286	27	0.386	2	0.029	10	0.143	13	0.186	2	0.029	12	0.171	15	0.214	0	0.000
Yugu (n = 96)	16	0.167	46	0.479	4	0.042	9	0.094	32	0.333	2	0.021	10	0.104	20	0.208	2	0.021

Fre.: Frequency.

**Table 6:** Distributions of KIR and HLA-C pairs in 11 ethnic populations.

	*2DL2/3*+*HLA-C1*		*2DL1*+*HLA-C2*		*2DS1*+*HLA-C2*		*2DS2*+*HLA-C1*	
	Counts	Fre.	Counts	Fre.	Counts	Fre.	Counts	Fre.
**Hani (n = 145)**	142	0.979	21	0.145	10	0.069	42	0.290
**Jinuo (n = 89)**	87	0.978	23	0.258	9	0.101	24	0.270
**Lisu (n = 99)**	98	0.990	16	0.162	5	0.051	33	0.333
**Nu (n = 106)**	104	0.981	22	0.208	4	0.038	11	0.104
**Bulang (n = 106)**	99	0.934	41	0.387	31	0.292	17	0.160
**Wa (n = 107)**	104	0.972	28	0.262	10	0.093	34	0.318
**Dai (n = 112)**	110	0.982	20	0.179	10	0.089	44	0.393
**Maonan (n = 82)**	81	0.988	6	0.073	2	0.024	35	0.427
**Zhuang (n = 95)**	95	1.000	21	0.221	11	0.116	28	0.295
**Tu (n = 70)**	62	0.886	31	0.443	11	0.157	22	0.314
**Yugu (n = 96)**	80	0.833	48	0.500	23	0.240	22	0.229

Fre.: Frequency.

## Supplementary Materials

The Supplementary Materials are available online at https://www.mdpi.com/2073-4409/8/7/711/s1.
