# Supplementary figures and images for: Distribution of Killer-Cell Immunoglobulin-Like Receptor Genes and Combinations of Their Human Leucocyte Antigen Ligands in 11 Ethnic Populations in China
Source: Cells. 2019 Jul 12;8(7):711. doi: 10.3390/cells8070711 (PMC6678321; doi:10.3390/cells8070711)

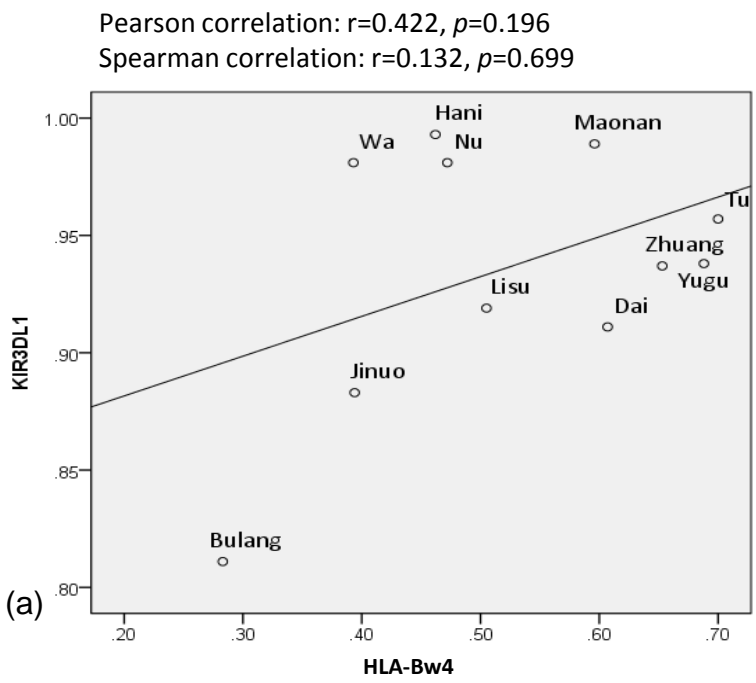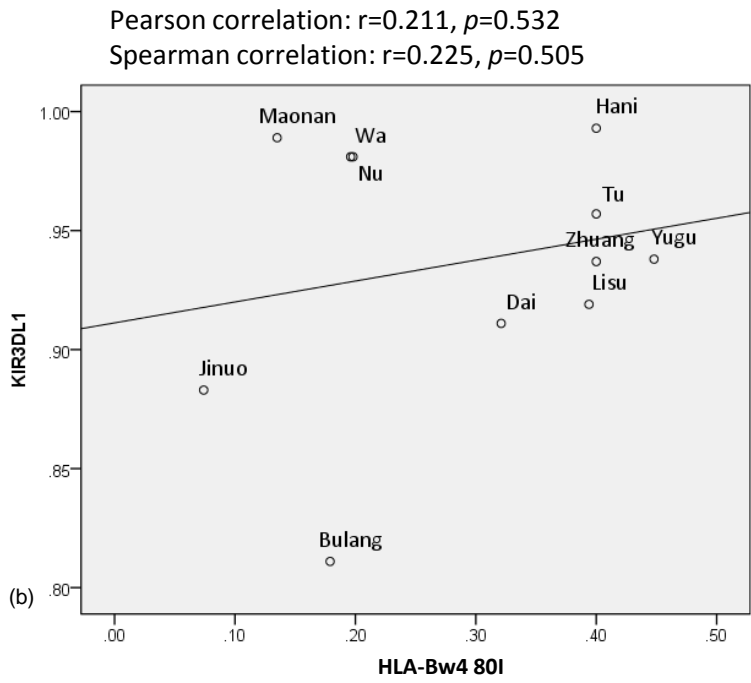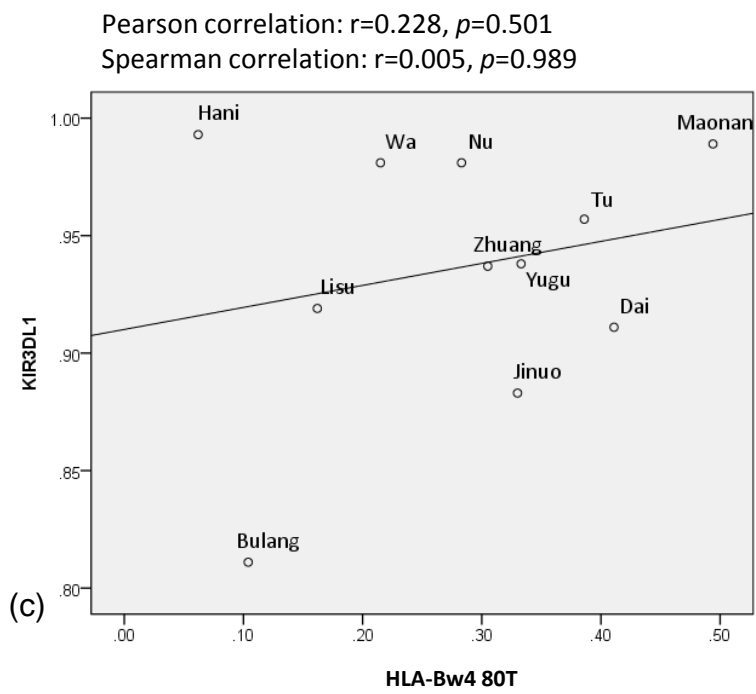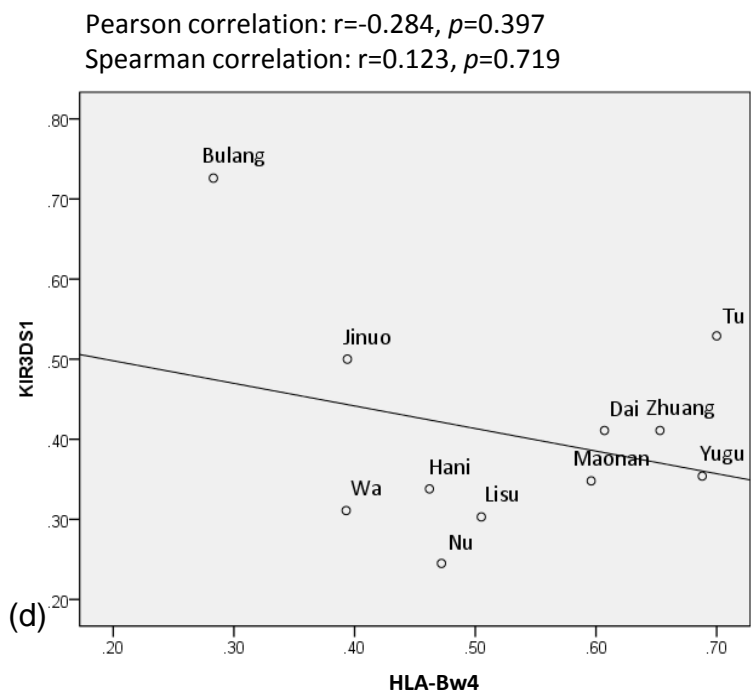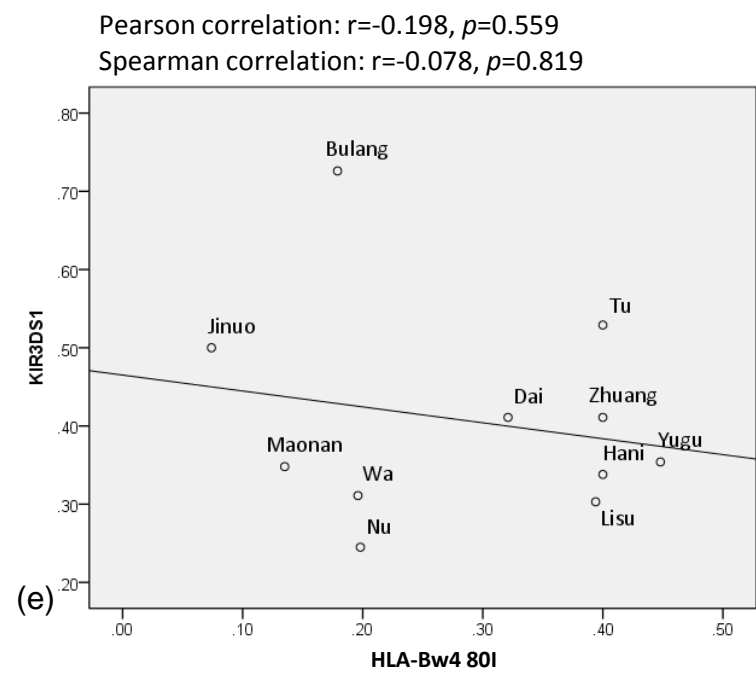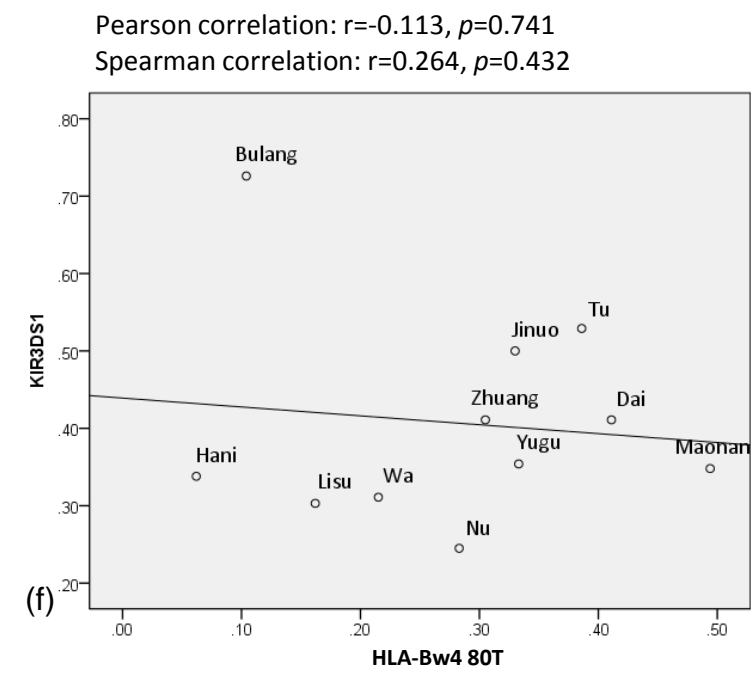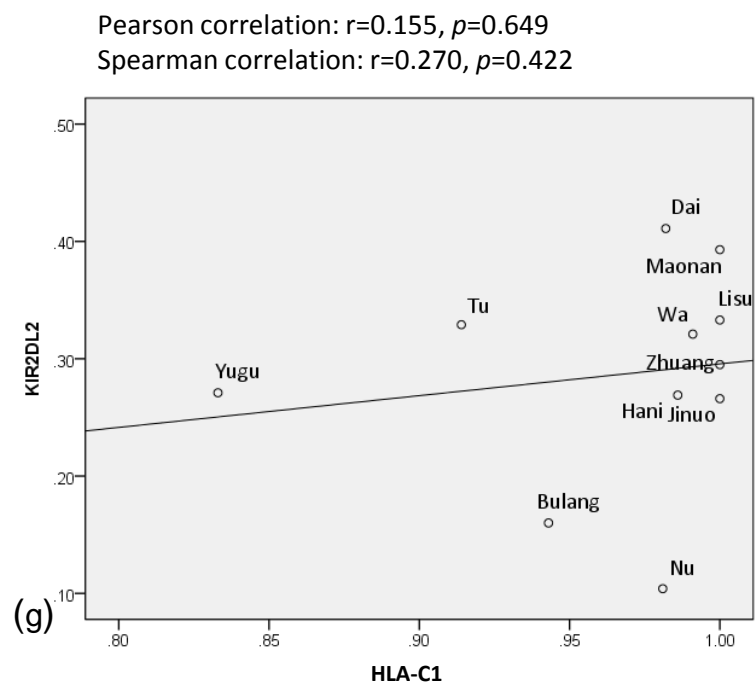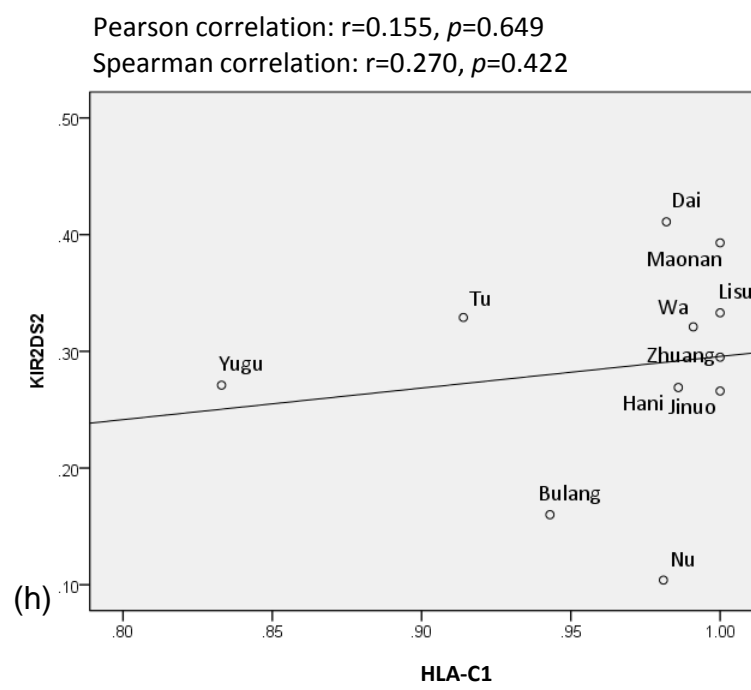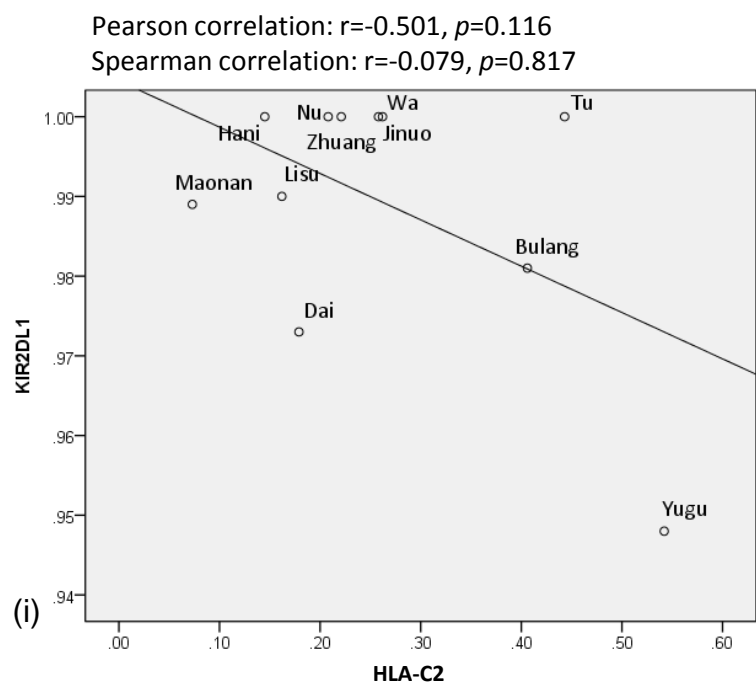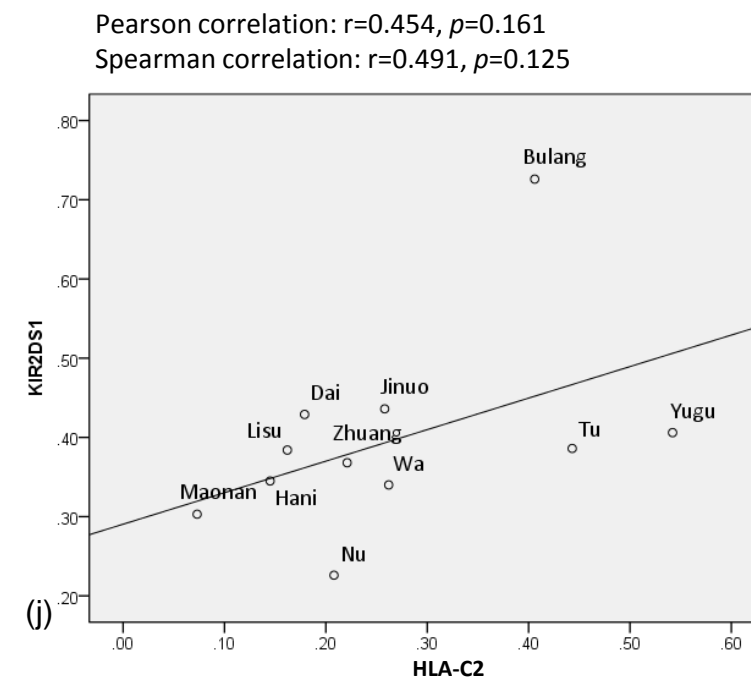

Supplement: Supplementary file 1 [file cells-08-00711-s001.zip › cells-528275-supplementary-final/supplementary files-R3/s-figure.pdf]
